# Annealing of Gadolinium-Doped Ceria (GDC) Films Produced by the Aerosol Deposition Method

**DOI:** 10.3390/ma11112072

**Published:** 2018-10-23

**Authors:** Jörg Exner, Hendrik Pöpke, Franz-Martin Fuchs, Jaroslaw Kita, Ralf Moos

**Affiliations:** 1 Department of Functional Materials, University of Bayreuth, Universitätsstraße 30, 95440 Bayreuth, Germany; 2 SOFC Department, Kerafol Keramische Folien GmbH, Koppe-Platz 1, 92676 Eschenbach, Germany

**Keywords:** dense films, electrical conductivity, thermal powder treatment, crystallite size, room temperature impact consolidation (RTIC)

## Abstract

Solid oxide fuel cells need a diffusion barrier layer to protect the zirconia-based electrolyte if a cobalt-containing cathode material like lanthanum strontium cobalt ferrite (LSCF) is used. This protective layer must prevent the direct contact and interdiffusion of both components while still retaining the oxygen ion transport. Gadolinium-doped ceria (GDC) meets these requirements. However, for a favorable cell performance, oxide ion conducting films that are thin yet dense are required. Films with a thickness in the sub-micrometer to micrometer range were produced by the dry room temperature spray-coating technique, aerosol deposition. Since commercially available GDC powders are usually optimized for the sintering of screen printed films or pressed bulk samples, their particle morphology is nanocrystalline with a high surface area that is not suitable for aerosol deposition. Therefore, different thermal and mechanical powder pretreatment procedures were investigated and linked to the morphology and integrity of the sprayed films. Only if a suitable pretreatment was conducted, dense and well-adhering GDC films were deposited. Otherwise, low-strength films were formed. The ionic conductivity of the resulting dense films was characterized by impedance spectroscopy between 300 °C and 1000 °C upon heating and cooling. A mild annealing occurred up to 900 °C during first heating that slightly increased the electric conductivity of GDC films formed by aerosol deposition.

## 1. Introduction

Solid oxide fuel cells (SOFC) are of high interest for clean and reliable energy conversion technologies. State of the art SOFCs based on ScSZ or YSZ (scandia- or yttria-stabilized zirconia) electrolytes and LSCF (lanthanum strontium cobalt ferrite) cathodes feature lower resistive losses compared to LSM (lanthanum strontium manganite) cathodes, especially at intermediate temperatures below 750 °C [1]. However, LSCF tends to react with YSZ or ScSZ during cell operation and fabrication. Therefore, a diffusion barrier layer is required to prevent the formation of insulating SrZrO_3_ [2]. For LSCF:YSZ based cells, oxide ion conducting gadolinium-doped ceria (GDC) can be used to separate cathode and electrolyte while retaining a low cell resistance. GDC has already been processed to thin films using e-beam evaporation [3] and to thick films using screen printing [4]. Conventionally sintered bulk samples were also produced [5]. However, since ceria is a high-temperature stable ceramic, high sintering temperatures, typically above 1200 °C, are required. For a high performance, mechanically stable GDC films with a high ionic conductivity are important. Both properties can be achieved by a novel spray-coating process known as the aerosol deposition method, usually abbreviated as AD or ADM. This dry spray-coating technique combines a variety of advantages over conventional coating methods. A key feature is the film deposition at room temperature, without the necessity of any heat treatment, neither during nor after the deposition. This means that dense ceramic films can be formed directly at room temperature just by spraying a sub-micrometer to micrometer-sized raw ceramic powder onto the surface to be coated. Film deposition and consolidation occurs by a process called room temperature impact consolidation (RTIC) that is based on fracturing and plastic deformation of impacting particles. Additionally, impacting particles consolidate previously deposited particles at the surface of the film (hammering effect [6]). Therefore, the crystallite size of the produced film is significantly decreased (at least by a factor of 10 to 15), compared to the sprayed ceramic powder [7,8]. As a consequence of the RTIC deposition mechanism, the sprayed films feature a very high, near theoretical density directly after deposition at room temperature, and already possess their ceramic properties like high hardness and wear resistance. A subsequent heat treatment (e.g., sintering) is not necessary. A more detailed description of the aerosol deposition process, its deposition mechanism, and the resulting film properties can be found in overview articles by Akedo’s group from 2008 [9] and, more recently, by our group from 2015 [10].

In this work, the formation of thin GDC films by the aerosol deposition method and their electrical properties are investigated. Since commercially available as-received GDC nanoparticles cannot be processed to films by AD, different powder pretreatment procedures were conducted in order to induce grain and particle growth as well as a mechanical activation. The dense yet thin (~1 µm) structure of aerosol deposited films combined with the deposition at room temperature could be especially advantageous over conventional screen-printed and sintered often porous GDC films.

## 2. Materials and Methods

**Figure 1:**
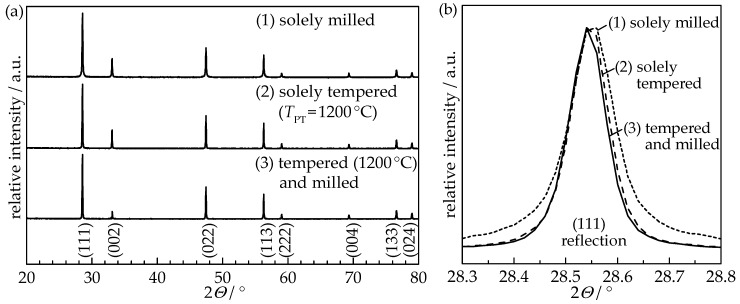
X-ray diffraction (XRD) patterns of pretreated GDC10 powders used for aerosol deposition: (**a**) complete 2*Θ* range from 20° to 80° and (**b**) (111) reflection at 2*Θ* = 28.5° (2*Θ* range from 28.3° to 28.8°). Note: GDC = gadolinium-doped ceria.

For all experiments, 10 mol-percent gadolinium-doped ceria nanopowder (GDC10, Treibacher Industrie AG, *d*_50_ < 0.4 µm, 99.9% purity) was used as a starting material. Three batches with different thermal and mechanical powder pretreatment procedures were produced: (1) Solely milled; (2) solely tempered at *T*_PT_ = 1200 °C; and (3) a combination of both using tempering at *T*_PT_ = 1200 °C followed by milling. In our previous publication, the minimum pretreatment temperature of 1100 °C for undoped ceria was found to enable successful aerosol deposition [8]. Since dopants may influence the thermal behavior (e.g., grain growth), a slightly increased temperature of 1200 °C was chosen for GDC10. Thermal powder pretreatment (tempering) was conducted in an air atmosphere in a muffle furnace. The pretreatment temperature *T*_PT_ of 1200 °C was held for 4 h with heating and cooling rates set to 5 K/min. Powders were milled in a planetary ball mill for 30 min using milling media and a jar made of zirconia with cyclohexane as milling fluid. Subsequently, cyclohexane was removed by a rotary evaporator and all three powders were furnace-dried at 120 °C for at least 48 h. Each powder was sieved (mesh size 90 µm) and dried at 200 °C for at least 24 h prior to deposition in order to obtain a free-flowing powder. X-ray diffraction (XRD) analysis (D8 Advance, Bruker, Billerica, MA, USA) using the X’Pert HighScore Plus software was implemented for structure verification and TOPAS-Academic software for Rietveld refinement with integrated algorithms for Williamson-Hall analysis to determine the crystallite size and the internal strain. Figure 1 shows the XRD patterns of all three pretreated GDC10 powders.

Rietveld refinement of the XRD pattern revealed an increase in crystallite size as a result of the powder annealing from 66 nm (solely milled powder) to 213 nm (tempered) and 210 nm (tempered and milled), respectively, as summarized in Table 1. Therefore, the annealing procedure at 1200 °C induced a significant change in the crystallographic structure of the GDC10 particles, while subsequent milling did not alter the crystallinity.

**Figure 2:**
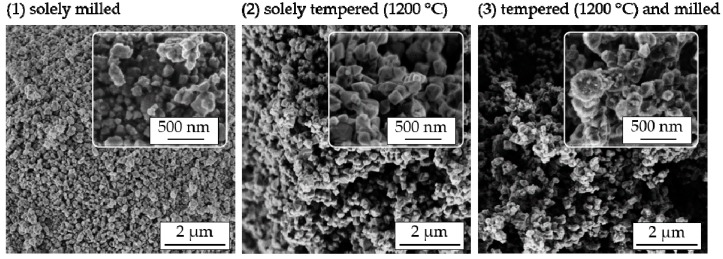
SEM images of GDC10 nanopowders after different powder pretreatments (insets use higher magnification): (**1**) Solely milled; (**2**) solely tempered at *T*_PT_ = 1200 °C; and (**3**) tempered at *T*_PT_ = 1200 °C and subsequently milled.

Furthermore, to investigate the influence of the powder pretreatment on the particle morphology, SEM imaging (Leo 1530 VP, Zeiss, Oberkochen, Germany) of all prepared powders was conducted, as displayed in Figure 2.

Powders that were solely milled (1) exhibit loosely connected nanoparticles with sizes between 100 nm and 300 nm. Through powder tempering at *T*_PT_ = 1200 °C (2), moderate grain and particle growth occurred (in accordance with the observed increase in crystallite size), leading to sharp-edged primary particles. Furthermore, these primary particles are connected by sintering necks to stable agglomerates larger than 5 µm. If a subsequent milling procedure was applied to tempered powders (3), sharp edges disappeared and primary particles developed a pronounced spherical shape again. Additionally, the agglomerate size decreased to 1–2 µm. Combining the information obtained from XRD and SEM, the following can be stated. After annealing at 1200 °C, and independent of the state of milling, powders consisted of 200 nm to 400 nm large primary particles whereas each primary particle consisted of one (single crystalline) or a few grains. The following milling procedure only influenced the size and morphology of hard agglomerates and to a small degree the shape of the primary particles.

A custom-made AD apparatus previously reported in [11] was used to produce the GDC10 films. Films with an area of 10 mm × 10 mm were deposited onto ScSZ substrates (scandia-stabilized zirconia, Kerafol Keramische Folien GmbH, Eschenbach, Germany) using a nozzle with an outlet slit-orifice size of 10 mm by 0.5 mm. During the coating process, the substrates were moved horizontally with a velocity of 2 mm/s. A 2 mm distance to the nozzle was adjusted, while the aerosol jet was formed by an oxygen carrier gas flow of 6 L/min. The pressures in the deposition chamber and in the aerosol generator were 1 mbar and 300 mbar, respectively. For electrical conductivity measurements, platinum interdigital electrodes (15 electrode fingers on each side with a width of 100 μm, a length of 4.7 mm, and a line spacing of 100 µm; for details see [12]) on an alumina substrate were also coated with a GDC10 film.

The coatings were evaluated with regard to their optical quality as well as their mechanical stability (tape adhesion test). The latter was conducted by neatly applying a scotch tape onto the deposited film so that nearly half of the sample surface (5 mm × 10 mm) was covered. The tape was smoothed and then removed by a fast, continuous motion. For “chalk-like” non-consolidated, low-strength films, the complete area delaminated and accumulated at the tape. For successfully sprayed films with a well-consolidated and dense microstructure, the film surface was not altered thus maintaining a clean tape. Therefore, this tape test can give a qualitative result regarding the adhesion and bonding strength of the deposited film. Additionally, XRD analysis of all the sprayed films as well as SEM imaging of one film were conducted to investigate the resulting film morphology and crystallographic structure.

## 3. Results and Discussion

**Figure 3:**
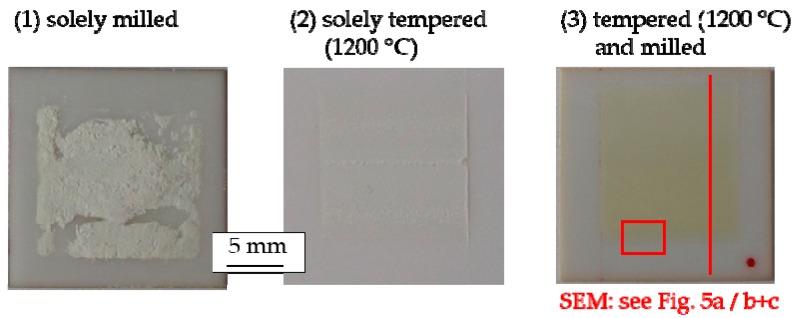
Optical quality of aerosol-deposited GDC10 films when differently pretreated powders were used: (**1**) Solely milled; (**2**) solely tempered at *T*_PT_ = 1200 °C; and (**3**) tempered at *T*_PT_ = 1200 °C and subsequently milled. The comparable images use identical scales.

Figure 3 depicts the optical appearance of the aerosol-deposited GDC10 films directly after the deposition.

The quality of sprayed AD films was highly dependent on the used pretreatment procedures. The solely milled powders built inhomogeneous, minimal strength films with uneven thicknesses. The films were completely removed by the tape test. The solely tempered GDC10 powder led to the deposition of a uniform film of around 10 µm. However, it still had not achieved the high integrity and stability of successfully aerosol-deposited films. Therefore, the film completely cracked and delaminated from the substrate during the tape test. On the contrary, AD of tempered (1200 °C) and subsequently milled powders resulted in high-strength films with superior adhesion to the substrate, film properties that are both well known for prosperous AD films [10]. For this film, no residue was visible at the tape.

**Figure 4:**
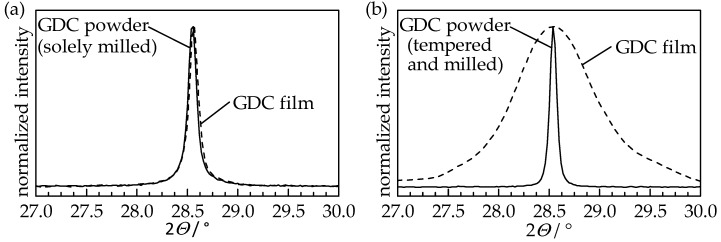
XRD patterns ((111) reflection within the 2*Θ* range from 27° to 30°) of GDC10 films formed by aerosol deposition and the therefore used GDC10 powders with different pretreatment procedures: (**a**) Solely milled and (**b**) tempered at *T*_PT_ = 1200 °C and subsequently milled.

To identify changes in the crystalline structure that happened during the aerosol deposition, XRD patterns of the GDC10 films were measured and compared to the therefore used GDC10 powders. Independent of the powder treatment, the cubic fluorite structure of the ceria powder was retained and is still present in the GDC10 films. In Figure 4, the (111) reflection of the solely milled powder and its aerosol-deposited film as well as the tempered (*T*_PT_ = 1200 °C) and milled powder and the resulting film are displayed.

The (111) reflection of the solely milled powder and the sprayed film thereof are identical, indicating that the impacted particles were not fractured but still exist in their initial shape. The solely tempered powder and its sprayed film exhibit the same behavior with a nearly matching reflex shape (therefore not shown in the Figure). However, when using the tempered and milled GDC10 powder, the reflections of the film are strongly broadened compared to the sharp shape of the powder. Using Rietveld refinement, this peak broadening is again attributed to the crystallite size and microstrain (for details of the Williamson-Hall method please see [8]). The crystallite sizes of chalk-like films, see Table 1, are similar to the used powders (solely milled as well as solely tempered), as already observed for undoped ceria [8], while the size in the stable film is reduced by a factor of 17.5 to 13 nm. On the contrary, a high microstrain of 0.67% is present in this stable GDC10 film, whereas chalk-like films possess no significant microstrain (less than 0.02%).

The decrease in crystallite size and the abrupt increase in microstrain are therefore necessary to deposit stable and well-adhering films that were only achieved for the tempered and milled GDC10 powder. This indicates that not only particles but especially crystallites fracture during the particle impact. It is assumed that the resulting fragments exhibit fresh, unoccupied, and therefore reactive surfaces. These fresh surfaces combined with the plastic deformation play an important role in the bonding mechanism of aerosol deposition [9,11]. The observed reduction in crystallite size was identified as crucial for the success of the deposition through RTIC [8].

**Figure 5:**
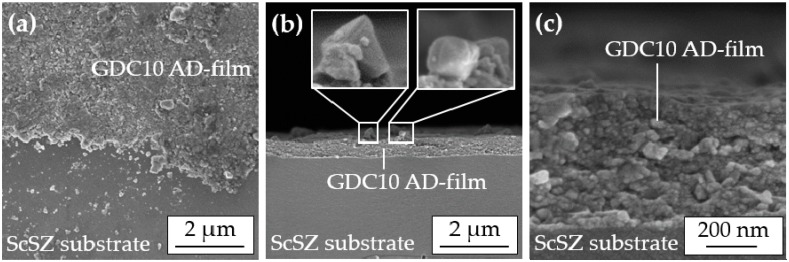
SEM images of a successfully sprayed GDC10 film using the tempered (*T*_PT_ = 1200 °C) and milled powder: (**a**) Top view near the edge of the coating and (**b**) fractured cross-section including loosely attached particles at the film surface (magnified inset); and (**c**) magnified film morphology.

SEM images of this successfully sprayed AD film reveal its superior quality, see Figure 5, with a fully dense (except for sporadic pores less than 20 nm in size), nanocrystalline morphology.

The top view image (a) clearly shows the homogeneous film without lateral pores. Cross-sectional images (b–c) indicate that no distinctive anchoring layer was formed due to the high hardness of the ScSZ substrate, in accordance with [13]. A noticeable amount of 200 nm to 400 nm large, loosely attached particles are visible on the surface of the AD film, enlarged by two insets. These particles still exist in a cuboid shape similar to the sprayed powder. This indicates that fracturing had not occurred there. Possibly, this particle morphology obstructs the aerosol deposition that could explain why solely tempered GDC10 powders with a pronounced cube-shape were not deposited to dense films.

**Figure 6:**
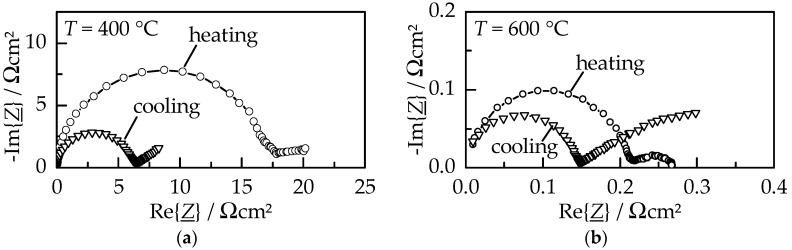
Normalized impedance spectroscopy data of a GDC10 film (with a thickness of 1.2 µm) on platinum interdigital electrodes (in air), upon initial heating and upon subsequent cooling (after a maximum temperature of 1000 °C). Shown are the normalized spectra at (**a**) 400 °C and (**b**) 600 °C.

The electrical behavior of a GDC10 film on platinum electrodes was characterized by impedance spectroscopy measurements in an alumina tubular furnace under air atmosphere. Normalized Nyquist plots upon heating and cooling within the first measured cycle at 400 °C and 600 °C are displayed in Figure 6.

**Figure 7:**
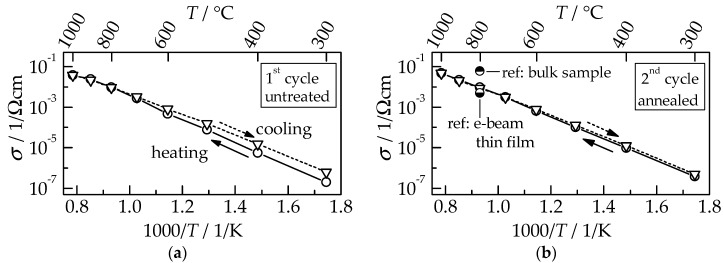
Electrical conductivity *σ* of the GDC10 film (with a thickness 1.2 µm) in an air atmosphere upon heating and subsequent cooling: (**a**) 1st cycle using the untreated, as-deposited film and (**b**) consecutive 2nd cycle of the identical film annealed at 1000 °C. Literature values at *T* = 800 °C are inserted in subfigure (**b**) for comparison (films by e-beam evaporation [3] and sintered bulk samples [5]).

The normalized spectra consist of two contributions, namely a large semicircle (resistor–capacitor circuit, RC) at higher frequencies representing the ionic conductivity and an incomplete, buckled semicircle (constant phase element, CPE) at lower frequencies. The latter can be attributed to a surface charge due to its high capacitance (close to 0.2 mF/cm^2^ [14,15]); therefore, it describes the contribution of the platinum electrode. At both displayed temperatures, the GDC10 film exhibits an increased resistance (diameter of the semicircle) during the initial heating compared to the subsequent cooling. As a consequence of the heat treatment up to 1000 °C, the area specific resistance (ASR) of the ionic conductivity diminish from 18 Ωcm^2^ to 6.5 Ωcm^2^ at 400 °C and from 0.27 Ωcm^2^ to 0.15 Ωcm^2^ at 600 °C, respectively. However, the resistance of the electrode contribution significantly increased, especially visible at 600 °C where the low-frequency CPE resistance is about a factor of ten larger during cooling. Although not completely understood yet, this behavior is thought to be connected to a reduction of the parasitic electronic film conductivity caused by a heat treatment, as observed by Schubert et al. for AD alumina films [16]. The ionic conductivity *σ* was calculated using the described geometry of the interdigital electrodes [17] and a GDC10 film thickness of 1.2 µm. An Arrhenius-like representation of *σ* is displayed in Figure 7 for the first (a) and consecutive second measuring cycle (b), each consisting of heating up to 1000 °C and subsequent cooling to 300 °C.

For the untreated GDC film within the first cycle, *σ* is slightly reduced during the first heating compared to cooling, as discussed for the Nyquist plots in Figure 7. This behavior occurs for most functional AD films during initial heating (annealing) [18,19,20]. Crystalline defects, as well as strain, that are formed by the RTIC process are released by this thermal annealing. At a temperature of 900 °C, the conductivity values of heating and cooling runs are equal, indicating that the annealing is completed. However, the total increase in conductivity by a factor of four, determined at 300 °C between the untreated and annealed state, is relatively small compared to the typically observed increases that range from 10 to 10^4^ [17,21,22]. In the consecutive second cycle, *σ* during heating and cooling coincides. The thermal activation energy of 1.0 eV is typical of polycrystalline GDC films [3]. While *σ* of AD films exceeds those of e-beam evaporated films [3], it is lower than the value for sintered bulk samples by a factor of six [5] (see ref. in Figure 7b). However, sintering requires high temperatures ranging from 1200 °C up to 1400 °C to be held for several hours to manufacture the samples, while aerosol deposition offers film deposition directly at room temperature. The thermal annealing of aerosol-deposited films to improve their ionic conductivity can be achieved during cell production when the subsequently added screen-printed NiO anode and LSCF cathode are sintered above 1000 °C. However, if anode and cathode are also manufactured using aerosol deposition, as was shown for cathode materials by Choi et al. for LSM and LSCF [23] and by Kim et al. for the NiO anode material [24], it would enable an SOFC to be produced completely at room temperature. Since typical SOFCs are operated at 850 °C, annealing of the aerosol-deposited GDC films to regain the high conductivity values could be achieved in situ during the first cell operation. Therefore, no additional heat treatment procedure would be necessary. Although the GDC10 diffusion barrier layer might not be necessary to avoid interdiffusion during the cell production at room temperature, it may ensure the favorable long-term application by minimizing the performance loss of the cell during operation.

The dense microstructure combined with a small thickness in the micrometer range in addition to high ionic conductivities make aerosol-deposited films an ideal diffusion barrier layer for ScSZ-based SOFC membranes.

## 4. Conclusions

Dense and well-adhering films of gadolinium-doped ceria (GDC) could be formed by the aerosol deposition method if a suitable powder pretreatment was applied. While solely milled as well as solely tempered (1200 °C) powders built low-strength films, a combination of tempering at 1200 °C and subsequent milling lead to the deposition of high-quality films with up to 1.2 µm thickness. The crystallite size of the powder was confirmed as a key parameter for successful aerosol deposition. The high densities and flawless morphologies of the GDC AD films were verified by SEM images. Impedance spectroscopy between 300 °C and 1000 °C revealed a slight annealing effect that increases the ionic conductivity up to a factor of four.

## Figures and Tables

**Table 1:** Crystallite sizes of pretreated powders and therefrom-sprayed aerosol deposited (AD) films.

	(1) Solely Milled	(2) Solely Tempered (1200 °C)	(3) Tempered (1200 °C) and Milled
Powder	66 nm	213 nm	210 nm
AD Film	61 nm	155 nm	13 nm

## References

[1] Haanappel V.A.C., Mai A., Mertens J. (2006). Electrode activation of anode-supported SOFCs with LSM- or LSCF-type cathodes. Solid State Ion..

[2] Paciejewska K.M., Yu Y., Kühn S., Weber A., Kleber M. (2015). Adjustment of GDC layer microstructure and its influence on the electrochemical properties of microtubular SOFCs. J. Ceram. Soc. Jpn..

[3] Zienius M., Bockute K., Virbukas D., Laukaitis G. (2017). Oxygen ion conductivity in samarium and gadolinium stabilized cerium oxide heterostructures. Solid State Ion..

[4] Jung H.-G., Sun Y.-K., Jung H.Y., Park J.S., Kim H.-R., Kim G.-H., Lee H.-W., Lee J.-H. (2008). Investigation of anode-supported SOFC with cobalt-containing cathode and GDC interlayer. Solid State Ion..

[5] Wang S., Kobayashi T., Dokiya M., Hashimoto T. (2000). Electrical and Ionic Conductivity of Gd-Doped Ceria. J. Electrochem. Soc..

[6] Lee D.-W., Kim H.-J., Nam S.-M. (2010). Effects of Starting Powder on the Growth of Al2O3 Films on Cu Substrates Using the Aerosol Deposition Method. J. Korean Phys. Soc..

[7] Akedo J. (2006). Aerosol Deposition of Ceramic Thick Films at Room Temperature: Densification Mechanism of Ceramic Layers. J. Am. Ceram. Soc..

[8] Exner J., Schubert M., Hanft D., Kita J., Moos R. (2018). How to treat powders for the room temperature aerosol deposition method to avoid porous, low strength ceramic films. J. Eur. Ceram. Soc..

[9] Akedo J. (2008). Room Temperature Impact Consolidation (RTIC) of Fine Ceramic Powder by Aerosol Deposition Method and Applications to Microdevices. J. Therm. Spray Technol..

[10] Hanft D., Exner J., Schubert M., Stöcker T., Fuierer P., Moos R. (2015). An Overview of the Aerosol Deposition Method: Process Fundamentals and New Trends in Materials Applications. J. Ceram. Sci. Technol..

[11] Exner J., Hahn M., Schubert M., Hanft D., Fuierer P., Moos R. (2015). Powder requirements for aerosol deposition of alumina films. Adv. Powder Technol..

[12] Exner J., Albrecht G., Schönauer-Kamin D., Kita J., Moos R. (2017). Pulsed Polarization-Based NOx Sensors of YSZ Films Produced by the Aerosol Deposition Method and by Screen-Printing. Sensors.

[13] Schubert M., Hahn M., Exner J., Kita J., Moos R. (2017). Effect of substrate hardness and surface roughness on the film formation of aerosol-deposited ceramic films. Funct. Mater. Lett..

[14] Boukamp B.A. (2008). Electrochemical Impedance Spectroscopy. Electrocatalysis@Nanoscale: Techniques and Applications, 24–28 December 2008.

[15] Irvine J.T.S., Sinclair D.C., West A.R. (1990). Electroceramics: Characterization by Impedance Spectroscopy. Adv. Mater..

[16] Schubert M., Leupold N., Exner J., Kita J., Moos R. (2018). High-Temperature Electrical Insulation Behavior of Alumina Films Prepared at Room Temperature by Aerosol Deposition and Influence of the Annealing Process and Powder Impurities. J. Therm. Spray Technol..

[17] Exner J., Fuierer P., Moos R. (2014). Aerosol deposition of (Cu,Ti) substituted bismuth vanadate films. Thin Solid Films.

[18] Ryu J., Park D.-S., Schmidt R. (2011). In-plane impedance spectroscopy in aerosol deposited NiMn2O4 negative temperature coefficient thermistor films. J. Appl. Phys..

[19] Baba S., Tsuda H., Akedo J. (2008). Thickness dependence of electrical properties of PZT films deposited on metal substrates by laser-assisted aerosol deposition. IEEE Trans. Ultrason. Ferroelect. Freq. Contr..

[20] Choi J.-J., Cho K.-S., Choi J.-H., Ryu J., Hahn B.-D., Kim J.-W., Ahn C.-W., Yoon W.-H., Yun J., Park D.-S. (2012). Effects of annealing temperature on solid oxide fuel cells containing (La,Sr)(Ga,Mg,Co)O3-δ electrolyte prepared by aerosol deposition. Mater. Lett..

[21] Exner J., Schubert M., Hanft D., Stöcker T., Fuierer P., Moos R. (2016). Tuning of the electrical conductivity of Sr(Ti,Fe)O3 oxygen sensing films by aerosol co-deposition with Al2O3. Sens. Actuators B.

[22] Khan A., Ahn C.-W., Ryu J., Yoon W.-H., Hahn B.-D., Choi J.-J., Kim J.-W., Park D.-S. (2014). Effect of Annealing on Properties of Lithium Aluminum Germanium Phosphate Electrolyte Thick Films Prepared by Aerosol Deposition. Met. Mater. Int..

[23] Choi J.-J., Lee J.-H., Park D.-S., Hahn B.-D., Yoon W.-H., Lin H.-T. (2007). Oxidation Resistance Coating of LSM and LSCF on SOFC Metallic Interconnects by the Aerosol Deposition Process. J. Am. Ceram. Soc..

[24] Kim H., Yang S., Ahn S.-H., Lee C.S. (2016). Effect of particle size on various substrates for deposition of NiO film via nanoparticle deposition system. Thin Solid Films.

